# Autologous Cell Seeding in Tracheal Tissue Engineering

**DOI:** 10.1007/s40778-017-0108-2

**Published:** 2017-10-26

**Authors:** Elizabeth F. Maughan, Robert E. Hynds, Toby J. Proctor, Sam M. Janes, Martin Elliott, Martin A. Birchall, Mark W. Lowdell, Paolo De Coppi

**Affiliations:** 1 Lungs for Living Research Centre, UCL Respiratory, University College London, London, UK; 2 Stem Cell and Regenerative Medicine Section, UCL Institute of Child Health and Great Ormond Street Hospital, 30 Guilford Street, London, WC1N 1EH UK; 3 Centre for Cell, Gene and Tissue Therapies, Royal Free Hospital & University College London, London, UK; 4 Great Ormond Street Hospital, London, UK; 5 UCL Centre for Regenerative Medicine, University College London, London, UK

**Keywords:** Trachea, Tissue engineering, Autologous cell seeding, Pre-clinical models, Clinical translation

## Abstract

**Purpose of Review:**

There is no consensus on the best technology to be employed for tracheal replacement. One particularly promising approach is based upon tissue engineering and involves applying autologous cells to transplantable scaffolds. Here, we present the reported pre-clinical and clinical data exploring the various options for achieving such seeding.

**Recent Findings:**

Various cell combinations, delivery strategies, and outcome measures are described. Mesenchymal stem cells (MSCs) are the most widely employed cell type in tracheal bioengineering. Airway epithelial cell luminal seeding is also widely employed, alone or in combination with other cell types. Combinations have thus far shown the greatest promise. Chondrocytes may improve mechanical outcomes in pre-clinical models, but have not been clinically tested. Rapid or pre-vascularization of scaffolds is an important consideration. Overall, there are few published objective measures of post-seeding cell viability, survival, or overall efficacy.

**Summary:**

There is no clear consensus on the optimal cell-scaffold combination and mechanisms for seeding*.* Systematic in vivo work is required to assess differences between tracheal grafts seeded with combinations of clinically deliverable cell types using objective outcome measures, including those for functionality and host immune response.

**Electronic supplementary material:**

The online version of this article (10.1007/s40778-017-0108-2) contains supplementary material, which is available to authorized users.

## Introduction

Treatment outcomes for long-segment tracheal disease in children have been steadily improving over the last decade, due mostly to advances in the slide tracheoplasty technique and improvements in post-operative care [1]. However, there remains a small subset of patients with extensive disease who cannot be managed by conventional means, as surgical resection would lead to an unacceptable level of tension on the anastomotic joins and failure of ventilation from kinking of the carina [2]. Endoscopic or conventional open surgery, such as end-to-end resection, can manage the majority of adult patients with acquired or idiopathic tracheal stenosis [3, 4]. These patients are those whose tracheal disease exceeds 50% of the total tracheal length in adults (or 30% of the total length in children) [5, 6], those whose primary reconstruction is unsuccessful, or those whose underlying tracheal disease recurs. In adults, voice outcomes from resection surgery are often suboptimal and recurrent stenosis is common [7]. In these, sometimes life-threatening and life-changing, circumstances, part- or whole-organ scale replacement of the trachea could be life-transforming or life-saving.

Interest in the field of regenerative medicine has grown exponentially in recent decades, and the subfield of tissue engineering sits at the intersection between cell biology, materials science, and engineering. In contrast to passive implants and medical constructs that are already widely employed across human and veterinary medicine, tissue-engineered medical devices aim to functionally repair, replace, or regenerate living tissue [8, 9]. The basic principle behind tissue engineering is to manufacture a biocompatible scaffold that supports the growth and differentiation of the recipient’s cells, to create a functioning neo-organ upon implantation [10]*.* Personalized scaffolds created in this way should not evoke conventional immune rejection responses and can thus be implanted without the need for immunosuppressive medication. In children, functional regeneration and remodeling of the replaced tissue might obviate the risk of the child outgrowing the transplant and needing serial re-transplantation.

The trachea was initially considered, perhaps naively, to be a convenient “starter organ” on which to concentrate tissue engineering efforts, due to its relatively simple tubular anatomy and “basic” primary function of passive air conduction to the lungs [11]. Given the lack of alternative treatment options in end-stage (often emergent) tracheal disease, the use of experimental therapies raises fewer potential ethical objections than in other clinical areas [12]. Regenerative approaches to tracheal reconstruction have, therefore, been in the forefront of the movement to create tissue-engineered solutions to organ and tissue replacement, and compassionate use clinical cases in both adults [13, 14] and in children [15, 16••] have been reported. However, at this stage, there remain significant scientific and surgical hurdles to wider testing in clinical trials and general clinical acceptance of this technology [17]. As such, it is vital that some criteria are internationally adopted to move this field forward. We propose the following criteria be considered for this purpose: (i) the internationally accumulated body of pre-clinical and clinical data is appraised and taken into account in the planning of future work, with adoption of an international registry; (ii) both basic and translational research should be carefully evaluated and should be constantly reviewed by independent peers; (iii) patients should be centralized into fewer centers to accumulate appropriate experience; (iv) careful, ethical patient selection is required, using alternative conventional techniques wherever these remain possible; and (v) once consensus is reached on the best strategy for scaffold procurement, preparation, and cell seeding, formal, ethically approved and nationally/internationally regulated clinical trials are required to demonstrate superiority over conventional treatments. An open-minded, collaborative, and constructive approach is required to improve airway repair and replacement in this rare patient group with life-changing and life-threatening structural airway abnormalities.

Here, we critically review recent advances in the field, focusing on the application of autologous cells in tissue-engineered tracheal replacements and, specifically, on the use of such constructs in clinically relevant in vivo orthotopic transplantation models.

## Scaffold Options for Tracheal Tissue Engineering

The ideal tissue-engineered scaffold for airway transplantation would be capable of promoting exogenous cell engraftment and endogenous cell ingrowth, proliferation, and appropriate differentiation, while maintaining a patent airway. Moreover, this scaffold must be able to promote the rapid establishment and support of vasculature, to allow cell survival and functional differentiation [12]. To date, two main scaffold strategies have been proposed in tracheal tissue engineering: (1) decellularized human cadaveric donor tissue or (2) synthetic scaffolds created de novo. The overriding principle behind the production and use of decellularized biological scaffolds from cadaveric tissue is the removal of all cellular components that are thought to be capable of eliciting an allo-rejection response if left behind. This may be achieved by physical, mechanical, and chemical methods [18–20] and is intended to preserve the underlying extracellular matrix (ECM), including its critical microanatomy, as well as its structural and signaling components. Cells are removed or their immunologically active proteins denatured [21–24] before the scaffold is seeded with autologous cells from the recipient, which, as “self,” are incapable of eliciting an immune response in the patient. Such scaffolds have been shown to retain a wealth of specific molecular cues and anchorage points to support patient cell ingrowth, survival, and differentiation [25]. There is also evidence that implanted decellularized tissue can downregulate local immune responses, leading to a “remodeling” of immune cell phenotypes [26]. The main drawbacks to the use of decellularized cadaveric tissue scaffolds are a continued reliance on donor organ supply, which may be ameliorated if animal sources are validated for use in the future, and the potential removal of ECM signals, be they physical or chemical, which are needed for constructive remodeling to take place [27]. Moreover, if the decellularization process substantially changes the biomechanical integrity of the scaffold, the endogenous remodeling by patient cells may be suboptimal since mesenchymal cell differentiation is known to be affected by substrate “stiffness” [28]. Additionally, there remains a possibility that despite decellularization, scaffolds can still elicit an adverse immune response, either in the form of damage-associated molecular pattern proteins (DAMPs) [29] or through retention of nucleic acid remnants [30] or donor MHC molecules [31].

The alternative “synthetic” strategy employs non-toxic materials, such as synthetic polymers, to create an entirely de novo scaffold [32]. Scaffolds made in this way are easy to manufacture and sterilize, carry few ethical concerns surrounding their supply, and they can be built to a patient’s personal measurements using pre-operative imaging or endoscopic examination. Composite scaffolds can be manufactured to allow separate niches suitable for multiple cell types within the graft [33]. Moreover, the scaffold material can be tailored with interconnected pores or mesh structures to allow vessel ingrowth and integration [34], and the scaffold can be decorated with molecular cues to encourage desirable processes such as angiogenesis. Published work to date examining the ability of autologous cells to attach to synthetic material has generally shown these synthetic scaffolds to be inferior to biologic scaffolds in this regard [32]. As a result, considerable efforts are being devoted to optimizing the production and biocompatibility of synthetic materials for tissue engineering.

As yet, there is no international consensus on the ideal biomaterial for supporting tracheal regeneration in vivo [25]. The results of pre-clinical in vivo orthotopic transplantation studies are difficult to compare between centers due to inevitable methodological differences arising from the development of these complex procedures. Furthermore, it has proven difficult to blind researchers to experimental groups in pre-clinical studies where they are directly compared, given their drastically different macroscopic and microscopic appearances. This has been a major issue, given the mostly qualitative level of data generated by endoscopic and histological inspection in most studies.

Published clinical data are limited to single, disparate, case reports, in which there are often unique and specific confounding issues associated with individual patient’s anatomical anomalies or co-morbidities. In such reports, it is impossible to establish a relationship between clinical outcomes and particular mechanistic processes from the use of specific cells and scaffolds. However, the synthetic strategy has recently largely fallen out of favor in Europe and the USA in the light of concerns raised regarding the conduct of the research reported in the synthetic case [35, 36]. Reports of poor scaffold integration parallel findings from other specialties that synthetic scaffolds used in the mediastinum can migrate to trachea and or esophagus [37, 38]. Present pre-clinical and clinical trial efforts in this field thus currently rely on the use of biologic tissue (decellularized or others, e.g. homografts), and the data thus far suggest these natural scaffolds exhibit superior safety and efficacy compared to their synthetic counterparts [20]. Innovative technologies like 3D printing, more reliable absorbable scaffolds (which are essential in pediatrics to allow remodeling with growth), and micro-definition of smart polymers containing adhesion molecules and specific growth factors, may one day allow us to move away from cadaveric organs and have access to a more reliable, “off-the-shelf” technology.

## Autologous Cell Choices for Tracheal Regeneration

The main property of an ideal tracheal replacement is the mechanical ability to withstand collapse under the physiological internal and external pressure changes of respiration. In addition, the proximal airways play an active role as the frontline defense against inhaled pathogens. The highly specialized pseudostratified ciliated epithelium, containing cells of both innate and adaptive immune systems, acts to trap and remove inhaled pathogens and debris. Overall, the current key goals in the field of tracheal replacement therapies are (1) to generate constructs that recapitulate native tracheal biomechanics, (2) to support a functional mucosa, and (3) to rapidly re-vascularize to ensure cell survival*.* While seeded somatic cells have not been shown to persist long-term within tissue-engineered grafts after transplantation, they seem to have an essential role in tissue remodeling and in vivo functional regeneration [39]. Unseeded scaffolds are more likely to develop strictures, granulations, and leakage and to provoke a local adverse immune response, which combine to ultimately lead to failure of the graft [40]. However, for the trachea and other “simple” hollow organs, it is still not entirely clear which cell types should be seeded before transplantation to assist functional regeneration of the engineered organ [41•]. This is particularly important for clinical translation, where the use of fewer cell types will greatly simplify production, substantially reduce product costs, and, theoretically, increase patient safety through facilitating the establishment of robust clinical manufacturing processes.

Reflective of the tissue composition of the native trachea, the three most widely studied autologous cell types for tracheal seeding in the pre-clinical setting (Appendix Table 1) are mesenchymal stromal/stem cells (MSC), chondrocytes, and epithelial cells.

Presently, there is no consensus as to the optimal cell and scaffold combination, as different cell combinations and seeding strategies have been reported in almost every pre-clinical and clinical series (Appendix Table 2)*.* We argue that to accurately conduct these comparisons, it is essential to establish appropriate in vivo models and for researchers in the field to reach an agreement as to the most meaningful objective outcome measures to be evaluated.

### Mesenchymal MSCs

Stromal/stem cells (MSCs) are a popular choice for recellularization in many tissue-engineered organ types [42] due to their ability to differentiate towards bone, fat, cartilage, and potentially other lineages [43], their production of wide-ranging trophic and immunomodulatory factors [44], and their ease of isolation and ability to be extensively expanded in vitro [45]. MSCs have predominantly been isolated from the bone marrow (BM-MSCs) [46], but those from a range of tissues, including adipose tissue [47], Wharton’s Jelly [48] and amniotic fluid [49], have been characterized. Little is known about human airway-resident MSCs, but human lung MSCs share a fundamental transcriptional profile with BM-MSCs, suggesting the latter may well be an appropriate surrogate in tissue engineering applications [50].

MSCs are thought to be somewhat immunoprivileged cells, and they possess potent immunosuppressive properties, particularly against allogeneic T cells [51]. These characteristics have led to the use of allogeneic MSCs in a number of clinical trials for a variety of therapeutic indications [52] and may also prove helpful for modulating host cell-mediated degradation of tissue-engineered scaffolds [53]. Clinical applications of MSCs have repeatedly demonstrated that these cells are safe [54]; while there are theoretical safety concerns that implanted MSCs might give rise to tumors in vivo, no evidence to support this theoretical risk has ever been seen in any pre-clinical or clinical assessments of MSCs [14].

There is increasing pre-clinical evidence to support the benefit of MSC seeding for tracheal tissue engineering, albeit in relatively small-scale studies. Suzuki et al. compared the use of allogeneic rat adipose-derived MSC-soaked collagen sponges compared to collagen alone in the repair of anterior tracheal defects in rats and found that the inclusion of MSCs accelerated vascularization and the regeneration of a well-organized epithelium in 2 weeks [55]. Batioglu-Karaaltin et al. compared decellularized tracheal transplantation with or without autologous adipose tissue-derived MSCs in rabbits, and whereas control animals suffered from early fatal graft separation, airway stenosis, and infection, animals receiving MSCs demonstrated only mild to moderate levels of stenosis, with complete graft integration at 3 months [56]. Clark et al. also found better integration of MSC-seeded grafts compared to cell-free control grafts [57]. It is important to note, however, that although autologous MSCs may be helpful in tissue integration and revascularization of grafts, they do not represent a solution for the recurring issues of anastomotic and graft stenosis that can plague orthotopic tracheal replacement [58].

Clinically, MSCs have been used as the main cell type for pre-implantation seeding in three published cases (one adult who received a synthetic graft [35, 36] and two children with decellularized grafts, one of which was prepared to GMP cell therapy standards) [15, 59••]. The relevance and source of the cellular contribution to the success of these grafts is difficult to dissect in the absence of formal clinical trials, so the relationship to pre-clinical studies involving MSCs is not clear.

### Chondrocytes

In pre-clinical studies, the most common site for harvesting differentiated chondrocytes is auricular elastic cartilage [60–67]. Despite the hyaline nature of tracheal rings, there is some evidence that the origin of chondrocyte does not affect the success of tracheal hyaline regeneration in pre-clinical small patch tracheoplasties [67–69]. Other groups have focused on the in vitro derivation of chondrocytes from cultures of MSCs [70, 71]. Embryonic stem cells can also differentiate into functional chondrocytes, but ethical concerns surrounding their use and their capacity for teratoma formation are significant hurdles to translational use. The ability to obtain differentiated chondrocytes from patient-specific induced pluripotent stem cells (iPSC) is potentially a more relevant avenue for exploration. Indeed, successful generation of hyaline cartilage in small animal models has been reported [72, 73]. Unfortunately, protocols for differentiating iPSC to chondrocytes remain rather limited at present [74, 75].

Outcome measures in studies involving chondrocyte seeding have included the persistence of chondrocytes within scaffolds for up to 14 weeks post-operatively [60, 69, 76–80] and the detection of appropriate extracellular matrix protein deposition, specifically collagen, elastin, and GAGs [62, 81]. Areas of “neocartilage” formation following chondrocyte seeding have been reported in several small pre-clinical studies of synthetic [60, 68, 78, 81] and decellularized [70] grafts, though few of these reports include unseeded control groups, making data interpretation somewhat difficult. Moreover, these reports have not included comments on the functionality of the new cartilage in contributing to the mechanical stability of grafts, despite the particular relevance of this in studies where scaffolds are manufactured from biodegradable material.

In the reported clinical literature, only one patient has received decellularized grafts containing chondrocytes derived from autologous MSCs that were culture expanded and differentiated in vitro [82]. Without deep biopsy of the implanted grafts, it is not possible to comment on the persistence or eventual fate of the seeded MSCs or chondrocytes nor the role these cells played in the overall graft success in such patients. Despite pre-clinical studies [60, 62, 63, 65, 67, 71, 76, 78, 80, 81, 83–86], to our knowledge, there have been no clinical reports of the use of harvested differentiated chondrocytes in tracheal tissue engineering.

### Epithelial Cells

Since MSCs are stromal and do not differentiate towards respiratory epithelial cells, they cannot restore barrier function or contribute directly to the regeneration of the mucociliary epithelium in tracheal grafts. Hence, many groups have investigated the possibility of enhancing tracheal regeneration by seeding the scaffolds with epithelial cells. A few pre-clinical studies have examined luminal epithelial cell seeding in the absence of other cell types [58, 87], but poor graft epithelialization was reported to lead to premature death from mucus plugging, pneumonia, or graft infection in the few studies in which epithelial cells were not co-seeded with MSCs [70, 83]. The restoration of a functional epithelium is clearly dependent on the surface area of coverage required. Short length or partial circumference grafts have been shown to epithelialize more quickly in both pre-clinical and clinical contexts, probably from ingrowth from the anastomotic margins [57, 86, 88–92]. In rabbit models, where the required surface area of an anterior graft is usually less than 5 cm^2^, epithelialization of grafts from anastomotic ingrowth has been reported to be underway by 2 weeks [93] and to be completed by 10–14 weeks [80, 94].

The rationale for including epithelial cells in tissue engineering approaches for the trachea is twofold. The first is to mediate long-term regeneration of the airway epithelium directly through engraftment. In some clinical cases, it has been practical to expand autologous airway epithelial cells in culture [35, 36, 59••, 95] or to include explant biopsies within grafts [15]; however, based on the length of time taken for epithelial regeneration in these patients [14, 96], now thought that the seeded epithelial cells were unlikely to have contributed to epithelial restoration. This is likely explained by the need for a vascular supply to be established prior to successful epithelial cell seeding, a hypothesis supported by the greater epithelialization in some pre-clinical studies employing periods of heterotopic pre-vascularization prior to orthotopic tracheal replacement [58, 60, 83]. Most clinical cases report the epithelialization process can take up to 2 years in segmental grafts, during which time patients remain vulnerable to mucus plugging and graft infection unless they receive frequent, inconvenient, and uncomfortable cleaning by bronchoscopy [15]. The presence of fibroblasts, known to improve epithelial survival and culture in vitro [97, 98], has been suggested to expedite this process in some in vivo animal studies in small sections of grafts [68, 76, 99–101]. It is hoped that improved understanding of human airway epithelial stem cell biology and robust techniques to apply cultured epithelial cells to grafts will ultimately move the field forward sufficiently to allow the development of epithelial cell therapies that are able to contribute directly to long-term regeneration.

The second argument for including epithelial cells is that these cells could be employed as a biological dressing, providing barrier function and signals to enhance local regeneration, rather than being required to engraft long term and contribute to tissue regeneration. Delaere and colleagues employed buccal mucosa grafts to cover the luminal surface of pre-implanted tracheal transplants following the degeneration of the donor mucosa on withdrawal of immunosuppression [102, 103]. While these cells are unlikely to transdifferentiate to produce ciliated epithelium, this mucosal source could prove helpful to create a temporary barrier to infection in extensive grafts, pending regeneration of respiratory mucosa from the anastomotic margins. Indeed, there is evidence from epidermal transplantation that such a “biologically active dressing” approach, using allogeneic [104] or even lyophilized [105] epithelial cells, can stimulate regeneration from surrounding host cells.

## Cell Seeding Considerations

It stands to reason that, given the complexity of the native trachea, it is likely that multiple cell types need to be present in an optimized bioengineered tracheal transplant. Since MSCs aid appropriate proliferation and differentiation of other cell types, such as chondrocytes [106], in vitro and also aid the migration of endogenous epithelium to cover grafts [107], they are a prime candidate for co-seeding strategies. Haykal et al. reported that the dual application of autologous MSCs and tracheal epithelial cells in a heterotopic pig model helped to preserve cartilage within decellularized tracheal matrix, where the MSCs evoked an immunomodulatory response, increasing the numbers of infiltrating regulatory T cells, as compared to decellularized matrix alone [108]. Tsao et al. co-transplanted chondrocytes and MSCs and found the deposition of type II collagen within biodegradable synthetic tracheal grafts was higher in co-transplanted animals than in animals receiving a single cell type or no cells [83]. In another study, Go et al. found that animals receiving decellularized grafts that were seeded with both chondrocytes and epithelial cells had lower levels of graft stenosis and bacterial contamination and increased overall survival compared to animals receiving bare or single cell type-seeded scaffolds [70]. Based on these preclinical studies showing a benefit for seeding with more than one cell type, many of the clinical cases reported to date have aimed to co-seed distinct external and luminal cell types [15, 35, 36, 59••, 92, 95].

The interpretation of data concerning the ideal cell type(s) to be included in tissue-engineered grafts is hampered by the lack of studies that have investigated critical variables such as the numbers of cells seeded or the methods used to propagate the cells ex vivo. As a result, the number of MSCs or chondrocytes required, the optimal techniques used to apply them to the scaffold to achieve seeding “success,” and the parameters by which “success” can be judged all remain unclear. When surveying the existing literature, the reported scaffold cell seeding density varies widely (for example, a range of 1 × 10^6^ cells/ml [109] to 5 × 10^10^ cells/ml [57] for bone marrow MSCs), and repeated or sequential seeding of scaffolds has rarely been attempted. Additionally, significant variation in cell seeding efficiency may further impact the final number of cells surviving and the type of cells present within scaffolds at the point of implantation [57]. For epithelial cells, in vitro data suggest that over 1 × 10^6^/cm^2^ human airway epithelial cells are required for uniform seeding of decellularized tracheal grafts when seeding from a cell suspension, suggesting that the clinically applied protocol using serum-free growth medium may be inadequate to generate sufficient cell numbers for long-segment human grafts [110]. While most studies to date have involved seeding cultured cells onto biomaterials as a cell suspension, this approach may need to be reconsidered in the case of the epithelium, where precedent from epidermal and corneal transplantation indicates the superiority of cultured cell sheets and the importance of stem cell retention during culture [111].

Technologies such as custom-designed bioreactors have been utilized in an effort to improve the efficacy of ex vivo seeding, including coverage, migration, and proliferation [35, 36, 59••, 82, 92]. These systems typically allow for separate seeding and media flow for extraluminal and intraluminal compartments [70, 109]. Most groups employing bioreactor systems have reported that optimal seeding occurs with a period of initial static seeding to allow cells to adhere to the scaffold surface, followed by a variable length of time with dynamic movement of either the graft, media, or both [56, 66, 67, 71, 86, 107]. However, to date, these in vitro parameters do not seem to necessarily translate to cell retention or demonstrable improvements in in vivo outcomes.

The degree to which the benefits of cell-seeded grafts are due to direct cell engraftment and retention versus the effect of the release of both free and exosome-contained chemotactic and trophic factors [112] from these cells remains unclear. In a subcutaneous heterotopic tracheal allograft model, it has been reported that repeated doses of MSCs might be effective in reducing both fibrosis and the extent of immune infiltrate, despite the failure of MSCs to persist long term within the scaffolds following implantation [113]. In these circumstances, signals might provide a stimulus for regeneration through immune modulation or by host cell recruitment.

## Graft Vascularization

Establishment of a blood supply is essential for any graft to permit the supply of nutrients to seeded cells and to native/endogenous cells growing into the tissue-engineered organ. A major surgical hurdle to tracheal replacement is that, unlike solid organs such as the kidney or lobes of the liver, there is no discrete blood supply that feeds the trachea; instead, the trachea relies on a finely segmental native blood supply, rather than a vascular pedicle that would be amenable to anastomosis. Bioengineered tracheal grafts are, therefore, relatively slow to establish vascular connections capable of supporting cell growth and tissue integration. The use of a period of heterotopic vascularization elsewhere in the body, prior to tracheal transfer as a pedicled or free flap transfer, has been shown to generate a clinically significant blood supply [60, 102]. Such a strategy may take several weeks––or even months––to become established and is therefore not possible in emergency situations. In such cases, an interposition graft or omental/pericardial vascularized wrap may be performed at the time of surgery, if such tissue is available [15, 91]. Pre-seeded cells may not survive the vascularization period, so delayed and/or multiple post-operative cell applications could be considered. Interestingly, autograft experiments in which animal tracheae have been removed (and in so doing, devascularized), inverted, and returned to the animal have resulted in minimal morbidity [114, 115], implying that the lack of a vascular pedicle is not necessarily a barrier to success if one can generate an optimized tracheal tissue-engineered scaffold.

## Limitations of Current Data

The pre-clinical and clinical literature regarding cell seeding in tracheal tissue engineering is a highly heterogeneous mix of experimental designs and outcome measures, which makes it difficult to provide clear recommendations for future autologous cell strategy choices. The pre-clinical studies identified here have often relied upon qualitative outcome measures such as histology, endoscopic observation, or the presence or absence of gross complications, rather than attempting to quantify cell retention, survival, or the differentiation of cells within the grafts. In addition to these limitations, there is rarely any mention in the literature as to blinding of either surgeons or researchers to the presence or absence of cells, or to randomization of animals. A particular challenge is demonstrating that cells found at post-mortem or biopsy are indeed those that were seeded pre-operatively, without some form of cell labeling to allow tracking. While transfection of cells to express a fluorescent protein [55, 66, 68, 99, 107, 109, 116] or the use of cell membrane dyes [83] has facilitated the tracking of seeded cells in some studies, these approaches do not rule out false-positive signals from dead, phagocytosed, or fused cells within the graft. Incorporation of luciferase into seeded cells––and the use of its substrate luciferin to generate a bioluminescent signal––would provide more compelling data, as this reaction can only occur in living cells [117]. Unfortunately, bioluminescence imaging is practically difficult in the medium to large animals that are most amenable to orthotopic transplantation and is certainly not practicable in human patients.

Care must be taken in assessing studies where small or only partial circumference grafts are employed, as ingrowth from endogenous tissue may lead to the over-reporting of benefit. Extrapolating such findings to model longer-segment segmental grafts, where circumferential granulation and stenosis remain unresolved hurdles in both pre-clinical and clinical scenarios [14], may not prove to be appropriate.

While the evolution of pre-clinical surgical techniques can be seen in many cases to model, as closely as possible, the clinical scenario in terms of endoscopic intervention and stent placement [65, 81, 118], it is still impractical to provide the level of intensive airway support and round-the-clock surveillance possible in a human intensive care setup. Most studies employ low numbers of individual animals in each experimental arm, and in many cases, a cell-free control arm has not been included. While this may represent well-meaning literal interpretation of the ethical pillars of animal research, namely to reduce the numbers of animals used as much as possible [119], the lack of inclusion of well-designed control groups greatly complicates meaningful analysis of results, given how many complex factors are in play in vivo. While seeded tracheal tissue-engineered grafts have been reported to outperform unseeded grafts in several pre-clinical studies [55, 56, 58, 70, 79, 80, 83, 100, 107, 116, 120], the most useful control arm to include to answer this question would be a scaffold with cell-conditioned medium only (given the wealth of growth factors present within most media). In addition, despite showing great promise, only a minority of pre-clinical studies have investigated and reported on the use of multiple cell types within the graft [70, 76, 83, 84, 99, 100].

Thoughtful and systematic experimental design of further animal studies will be vital to allow the testing of cell types and combinations, ratios, and seeding timings as objectively as possible [121]. In the clinical scenario, it is vital that international groups working in this field report their outcomes in as transparent a manner as possible, both to secure public trust in the integrity of the field and to allow groups who are undertaking such compassionate use treatments elsewhere to learn from their experiences [122•].

**Table:** 

Key proposals for international debate
1. Limited menu of pre-clinical animal in vivo experimental designs
2. Standard definitions of cells, and scaffolds and key criteria for reporting on results
3. Randomization and blinding of in vivo experimental and control arms
4. Internationally agreed objective outcome measures, e.g., epithelialization, functional cartilage formation, or degree of stenosis
5. International transparent register of tracheal tissue engineering clinical cases

## Conclusion

The incorporation of recipient cells into tissue-engineered tracheal replacement therapies appears crucial for graft survival and, once optimized, the inclusion of exogenous cells could directly improve graft functionality. While there is evidence that seeding of tracheal grafts with autologous cells is beneficial overall, particularly with regard to the use of MSCs, it is still unclear which specific cell types and combinations are optimal for promoting regeneration. Further, there are fundamental questions that remain unanswered regarding the optimal cell type(s) and seeding strategies in this context. It is clear that future pre-clinical studies should undergo rigorous and systematic experimental design to maximize the generation of clinically useful data regarding the roles of seeded cells, particularly with respect to graft mechanical stability, cartilage maintenance, epithelialization, stenosis, and vascularization.

## Electronic Supplementary Material

ESM 1 (DOCX 35.7 kb).

ESM 2 (DOCX 86.9 kb).

## References

[1] Butler CR, Speggiorin S, Rijnberg FM, Roebuck DJ, Muthialu N, Hewitt RJ (2014). Outcomes of slide tracheoplasty in 101 children: a 17-year single-center experience. J Thorac Cardiovasc Surg.

[2] Rutter MJ, Prosser JD. Congenital tracheal stenosis. In: Lioy J, Sobol SE, editors. Disorders of the Neonatal Airway. New York: Springer; 2015. p. 81–6.

[3] Nouraei SAR, Ghufoor K, Patel A, Ferguson T, Howard DJ, Sandhu GS (2007). Outcome of endoscopic treatment of adult postintubation tracheal stenosis. Laryngoscope.

[4] Grillo HC, Mathisen DJ (1990). Primary tracheal tumors: treatment and results. Ann Thorac Surg.

[5] Jacobs JP, Elliott MJ, Haw MP, Bailey CM, Herberhold C (1996). Pediatric tracheal homograft reconstruction: a novel approach to complex tracheal stenoses in children. J Thorac Cardiovasc Surg.

[6] Grillo HC (2002). Tracheal replacement: a critical review. Ann Thorac Surg.

[7] Clunie GM, Kinshuck AJ, Sandhu GS, Roe JW (2017). Voice and swallowing outcomes for adults undergoing reconstructive surgery for laryngotracheal stenosis. Curr Opin Otolaryngol Head Neck Surg.

[8] Atala A, Kasper FK, Mikos AG (2012). Engineering complex tissues. Sci Transl Med.

[9] Langer R, Vacanti JP (1993). Tissue engineering. Science.

[10] Maughan E, Birchall M (2014). Regenerated airways––opening transplantation up to a cancer population. Oncol News.

[11] Delaere PR, Van Raemdonck D (2014). The trachea: the first tissue-engineered organ?. J Thorac Cardiovasc Surg.

[12] Maughan E, Lesage F, Butler CR, Hynds RE, Hewitt R, Janes SM, et al., editors. Airway tissue engineering for congenital laryngotracheal disease. Seminars in Pediatric Surgery. Elsevier; 2016. 10.1053/j.sempedsurg.2016.02.012 27301606

[13] Crowley C, Birchall M, Seifalian AM (2015). Trachea transplantation: from laboratory to patient. J Tissue Eng Regen Med.

[14] Gonfiotti A, Jaus MO, Barale D, Baiguera S, Comin C, Lavorini F (2014). The first tissue-engineered airway transplantation: 5-year follow-up results. Lancet.

[15] Elliott MJ, De Coppi P, Speggiorin S, Roebuck D, Butler CR, Samuel E (2012). Stem-cell-based, tissue engineered tracheal replacement in a child: a 2-year follow-up study. Lancet.

[16] Hamilton NJ, Kanani M, Roebuck DJ, Hewitt RJ, Cetto R, Culme-Seymour EJ (2015). Tissue-engineered tracheal replacement in a child: a 4-year follow-up study. Am J Transplant.

[17] Russell AJ (2014). The end of the beginning for tissue engineering. Lancet.

[18] Conconi MT, Coppi PD, Liddo RD, Vigolo S, Zanon GF, Parnigotto PP (2005). Tracheal matrices, obtained by a detergent-enzymatic method, support in vitro the adhesion of chondrocytes and tracheal epithelial cells. Transpl Int.

[19] Jungebluth P, Go T, Asnaghi A, Bellini S, Martorell J, Calore C (2009). Structural and morphologic evaluation of a novel detergent–enzymatic tissue-engineered tracheal tubular matrix. J Thorac Cardiovasc Surg.

[20] Crapo PM, Gilbert TW, Badylak SF (2011). An overview of tissue and whole organ decellularization processes. Biomaterials.

[21] Badylak SF, Gilbert TW, editors. Immune response to biologic scaffold materials. Seminars in immunology. Elsevier; 2008. 10.1016/j.smim.2007.11.003 PMC2605275 18083531

[22] Saxena AK (2005). Tissue engineering: present concepts and strategies. J Indian Assoc Pediatr Surg.

[23] Fuchs JR, Nasseri BA, Vacanti JP (2001). Tissue engineering: a 21st century solution to surgical reconstruction. Ann Thorac Surg.

[24] Ott LM, Weatherly RA, Detamore MS (2011). Overview of tracheal tissue engineering: clinical need drives the laboratory approach. Ann Biomed Eng.

[25] Badylak SF, Freytes DO, Gilbert TW (2009). Extracellular matrix as a biological scaffold material: structure and function. Acta Biomater.

[26] Fishman JM, Lowdell MW, Urbani L, Ansari T, Burns AJ, Turmaine M (2013). Immunomodulatory effect of a decellularized skeletal muscle scaffold in a discordant xenotransplantation model. Proc Natl Acad Sci.

[27] Partington L, Mordan N, Mason C, Knowles J, Kim H, Lowdell M, et al. Biochemical changes caused by decellularization may compromise mechanical integrity of tracheal scaffolds. Acta Biomater. 2013;9(2):5251–61. 10.1016/j.actbio.2012.10.004 23059415

[28] Engler AJ, Sen S, Sweeney HL, Discher DE (2006). Matrix elasticity directs stem cell lineage specification. Cell.

[29] Daly K, Liu S, Agrawal V, Brown B, Johnson S, Medberry C (2012). Damage associated molecular patterns within xenogeneic biologic scaffolds and their effects on host remodeling. Biomaterials.

[30] Li Q, Uygun BE, Geerts S, Ozer S, Scalf M, Gilpin SE (2016). Proteomic analysis of naturally-sourced biological scaffolds. Biomaterials.

[31] Wiles K, Fishman JM, De Coppi P, Birchall MA (2016). The host immune response to tissue-engineered organs: current problems and future directions. Tissue Eng B Rev.

[32] Lutolf M, Hubbell J (2005). Synthetic biomaterials as instructive extracellular microenvironments for morphogenesis in tissue engineering. Nat Biotechnol.

[33] Panadero J, Lanceros-Mendez S, Ribelles JG (2016). Differentiation of mesenchymal stem cells for cartilage tissue engineering: individual and synergetic effects of three-dimensional environment and mechanical loading. Acta Biomater.

[34] Crowley C, Klanrit P, Butler CR, Varanou A, Platé M, Hynds RE (2016). Surface modification of a POSS-nanocomposite material to enhance cellular integration of a synthetic bioscaffold. Biomaterials.

[35] Jungebluth P, Alici E, Baiguera S, Le Blanc K, Blomberg P, Bozoky B (2011). Tracheobronchial transplantation with a stem-cell-seeded bioartificial nanocomposite: a proof-of-concept study. Lancet.

[36] The Lancet E. Expression of concern––tracheobronchial transplantation with a stem-cell-seeded bioartificial nanocomposite: a proof-of-concept study. Lancet. 387:1359. 10.1016/S0140-6736(16)30091-5 27115801

[37] Müller-Stich B, Senft J, Lasitschka F, Shevchenko M, Billeter A, Bruckner T (2014). Polypropylene, polyester or polytetrafluoroethylene—is there an ideal material for mesh augmentation at the esophageal hiatus? Results from an experimental study in a porcine model. Hernia.

[38] Porziella V, Cesario A, Lococo F, Margaritora S, Leuzzi G, Marchese M (2012). Complete transmural gastric migration of PTFE mesh after surgery for a recurrent hiatal hernia. Eur Rev Med Pharmacol Sci.

[39] Roh JD, Sawh-Martinez R, Brennan MP, Jay SM, Devine L, Rao DA (2010). Tissue-engineered vascular grafts transform into mature blood vessels via an inflammation-mediated process of vascular remodeling. Proc Natl Acad Sci.

[40] Hibino N, Yi T, Duncan DR, Rathore A, Dean E, Naito Y (2011). A critical role for macrophages in neovessel formation and the development of stenosis in tissue-engineered vascular grafts. FASEB J.

[41] Chiang T, Pepper V, Best C, Onwuka E, Breuer CK (2016). Clinical translation of tissue engineered trachea grafts. Ann Otol Rhinol Laryngol.

[42] Banas A, Teratani T, Yamamoto Y, Tokuhara M, Takeshita F, Quinn G (2007). Adipose tissue-derived mesenchymal stem cells as a source of human hepatocytes. Hepatology.

[43] Bianco P, Robey PG, Simmons PJ (2008). Mesenchymal stem cells: revisiting history, concepts, and assays. Cell Stem Cell.

[44] Caplan AI. Mesenchymal stem cells: time to change the name!. Stem Cells Transl Med. 2017;6(6):1445–51. 10.1002/sctm.17-0051 PMC5689741 28452204

[45] Kern S, Eichler H, Stoeve J, Klüter H, Bieback K (2006). Comparative analysis of mesenchymal stem cells from bone marrow, umbilical cord blood, or adipose tissue. Stem Cells.

[46] Pittenger MF, Mackay AM, Beck SC, Jaiswal RK, Douglas R, Mosca JD (1999). Multilineage potential of adult human mesenchymal stem cells. Science.

[47] Zuk PA, Zhu M, Mizuno H, Huang J, Futrell JW, Katz AJ (2001). Multilineage cells from human adipose tissue: implications for cell-based therapies. Tissue Eng.

[48] Wang HS, Hung SC, Peng ST, Huang CC, Wei HM, Guo YJ (2004). Mesenchymal stem cells in the Wharton’s jelly of the human umbilical cord. Stem Cells.

[49] De Coppi P, Bartsch G, Siddiqui MM, Xu T, Santos CC, Perin L (2007). Isolation of amniotic stem cell lines with potential for therapy. Nat Biotechnol.

[50] Sinclair K, Yerkovich S, Chen T, McQualter J, Hopkins P, Wells C (2016). Mesenchymal stromal cells are readily recoverable from lung tissue, but not the alveolar space, in healthy humans. Stem Cells.

[51] De Miguel MP, Fuentes-Julian S, Blazquez-Martinez A, Pascual CY, Aller MA, Arias J (2012). Immunosuppressive properties of mesenchymal stem cells: advances and applications. Curr Mol Med.

[52] Beyth S, Borovsky Z, Mevorach D, Liebergall M, Gazit Z, Aslan H (2005). Human mesenchymal stem cells alter antigen-presenting cell maturation and induce T-cell unresponsiveness. Blood.

[53] Yanez R, Lamana ML, García-Castro J, Colmenero I, Ramirez M, Bueren JA (2006). Adipose tissue-derived mesenchymal stem cells have in vivo immunosuppressive properties applicable for the control of the graft-versus-host disease. Stem Cells.

[54] Parekkadan B, Milwid JM (2010). Mesenchymal stem cells as therapeutics. Annu Rev Biomed Eng.

[55] Suzuki T, Kobayashi K, Tada Y, Suzuki Y, Wada I, Nakamura T (2008). Regeneration of the trachea using a bioengineered scaffold with adipose-derived stem cells. Ann Otol Rhinol Laryngol.

[56] Batioglu-Karaaltin A, Karaaltin MV, Ovali E, Yigit O, Kongur M, Inan O (2015). In vivo tissue-engineered allogenic trachea transplantation in rabbits: a preliminary report. Stem Cell Rev Rep.

[57] Clark ES, Best C, Onwuka E, Sugiura T, Mahler N, Bolon B (2016). Effect of cell seeding on neotissue formation in a tissue engineered trachea. J Pediatr Surg.

[58] Kanzaki M, Yamato M, Hatakeyama H, Kohno C, Yang J, Umemoto T (2006). Tissue engineered epithelial cell sheets for the creation of a bioartificial trachea. Tissue Eng.

[59] Elliot MJ, Butler CR, Varanou-Jenkins A, Parting ton L, Carvalho C, Samuel E (2017). Tracheal replacement therapy with a stem cell-seeded graft: lessons from compassionate use application of a GMP-compliant tissue-engineered medicine. Stem Cells Transl Med.

[60] Komura M, Komura H, Otani Y, Suzuki K, Satake R, Kodaka T (2015). Tracheoplasty with cartilage-engineered esophagus environments. J Pediatr Surg.

[61] Chang JW, Park SA, Park JK, Choi JW, Kim YS, Shin YS (2014). Tissue-engineered tracheal reconstruction using three-dimensionally printed artificial tracheal graft: preliminary report. Artif Organs.

[62] Luo X, Liu Y, Zhang Z, Tao R, Liu Y, He A (2013). Long-term functional reconstruction of segmental tracheal defect by pedicled tissue-engineered trachea in rabbits. Biomaterials.

[63] Gilpin DA, Weidenbecher MS, Dennis JE (2010). Scaffold-free tissue-engineered cartilage implants for laryngotracheal reconstruction. Laryngoscope.

[64] Kim DY, Pyun J, Choi JW, Kim JH, Lee JS, Shin H (2010). Tissue-engineered allograft tracheal cartilage using fibrin/hyaluronan composite gel and its in vivo implantation. Laryngoscope.

[65] Weidenbecher M, Tucker HM, Gilpin DA, Dennis JE (2009). Tissue-engineered trachea for airway reconstruction. Laryngoscope.

[66] Kunisaki SM, Freedman DA, Fauza DO (2006). Fetal tracheal reconstruction with cartilaginous grafts engineered from mesenchymal amniocytes. J Pediatr Surg.

[67] Lee C, Moon K, Choi H (2002). Tissue engineered tracheal prosthesis with acceleratedly cultured homologous chondrocytes as an alternative of tracheal reconstruction. Journal of. Cardiovasc Surg.

[68] Nomoto Y, Kobayashi K, Tada Y, Wada I, Nakamura T, Omori K (2008). Effect of fibroblasts on epithelial regeneration on the surface of a bioengineered trachea. Ann Otol Rhinol Laryngol.

[69] Fuchs JR, Terada S, Ochoa ER, Vacanti JP, Fauza DO (2002). Fetal tissue engineering: in utero tracheal augmentation in an ovine model. J Pediatr Surg.

[70] Go T, Jungebluth P, Baiguero S, Asnaghi A, Martorell J, Ostertag H (2010). Both epithelial cells and mesenchymal stem cell–derived chondrocytes contribute to the survival of tissue-engineered airway transplants in pigs. J Thorac Cardiovasc Surg.

[71] Fuchs JR, Hannouche D, Terada S, Vacanti JP, Fauza DO (2003). Fetal tracheal augmentation with cartilage engineered from bone marrow-derived mesenchymal progenitor cells. J Pediatr Surg.

[72] Diekman BO, Christoforou N, Willard VP, Sun H, Sanchez-Adams J, Leong KW (2012). Cartilage tissue engineering using differentiated and purified induced pluripotent stem cells. Proc Natl Acad Sci.

[73] Zhu Y, Wu X, Liang Y, Gu H, Song K, Zou X (2016). Repair of cartilage defects in osteoarthritis rats with induced pluripotent stem cell derived chondrocytes. BMC Biotechnol.

[74] Diederichs S, Gabler J, Autenrieth J, Kynast KL, Merle C, Walles H (2016). Differential regulation of SOX9 protein during chondrogenesis of induced pluripotent stem cells versus mesenchymal stromal cells: a shortcoming for cartilage formation. Stem Cells Dev.

[75] Cheng A, Hardingham TE, Kimber SJ (2013). Generating cartilage repair from pluripotent stem cells. Tissue Eng B Rev.

[76] Kojima K, Bonassar LJ, Roy AK, Vacanti CA, Cortiella J (2002). Autologous tissue-engineered trachea with sheep nasal chondrocytes. J Thorac Cardiovasc Surg.

[77] Yan B, Zhang Z, Wang X, Ni Y, Liu Y, Liu T, et al. PLGA–PTMC–cultured bone mesenchymal stem cell scaffold enhances cartilage regeneration in tissue-engineered tracheal transplantation. Artif Organs. 2017;41(5):461–9. 10.1111/aor.12805 27925229

[78] Hong HJ, Chang JW, Park JK, Choi JW, Kim YS, Shin YS (2014). Tracheal reconstruction using chondrocytes seeded on a poly (l-lactic-co-glycolic acid)–fibrin/hyaluronan. J Biomed Mater Res A.

[79] Kim H (2013). Influence of mesenchymal stem cells on cryopreserved tracheal allografts in rabbits. Korean J Thorac Cardiovasc Surg.

[80] Nomoto M, Nomoto Y, Tada Y, Tani A, Otsuki K, Suzuki R (2013). Bioengineered trachea using autologous chondrocytes for regeneration of tracheal cartilage in a rabbit model. Laryngoscope.

[81] Grimmer JF, Gunnlaugsson CB, Alsberg E, Murphy HS, Kong HJ, Mooney DJ (2004). Tracheal reconstruction using tissue-engineered cartilage. Arch Otolaryngol–Head Neck Surg.

[82] Macchiarini P, Jungebluth P, Go T, Asnaghi MA, Rees LE, Cogan TA (2008). Clinical transplantation of a tissue-engineered airway. Lancet.

[83] Tsao C-K, Ko C-Y, Yang S-R, Yang C-Y, Brey EM, Huang S (2014). An ectopic approach for engineering a vascularized tracheal substitute. Biomaterials.

[84] Jungebluth P, Bader A, Baiguera S, Moller S, Jaus M, Lim ML (2012). The concept of in vivo airway tissue engineering. Biomaterials.

[85] Shin YS, Lee BH, Choi JW, Min B-H, Chang JW, Yang SS (2014). Tissue-engineered tracheal reconstruction using chondrocyte seeded on a porcine cartilage-derived substance scaffold. Int J Pediatr Otorhinolaryngol.

[86] Lin C-H, Hsu S-H, Huang C-E, Cheng W-T, Su J-M (2009). A scaffold-bioreactor system for a tissue-engineered trachea. Biomaterials.

[87] Wood MW, Murphy SV, Feng X, Wright SC (2014). Tracheal reconstruction in a canine model. Otolaryngology––head and neck. Surgery.

[88] Kanemaru S-I, Hirano S, Umeda H, Yamashita M, Suehiro A, Nakamura T (2010). A tissue-engineering approach for stenosis of the trachea and/or cricoid. Acta Otolaryngol Suppl.

[89] Omori K, Nakamura T, Kanemaru S, Asato R, Yamashita M, Tanaka S (2005). Regenerative medicine of the trachea: the first human case. Ann Otol Rhinol Laryngol.

[90] Omori K, Tada Y, Suzuki T, Nomoto Y, Matsuzuka T, Kobayashi K (2008). Clinical application of in situ tissue engineering using a scaffolding technique for reconstruction of the larynx and trachea. Ann Otol Rhinol Laryngol.

[91] Macchiarini P, Walles T, Biancosino C, Mertsching H (2004). First human transplantation of a bioengineered airway tissue. J Thorac Cardiovasc Surg.

[92] Steinke M, Dally I, Friedel G, Walles H, Walles T (2014). Host-integration of a tissue-engineered airway patch: two-year follow-up in a single patient. Tissue Eng A.

[93] Okano W, Nomoto Y, Wada I, Kobayashi K, Miyake M, Nakamura T (2010). Bioengineered trachea with fibroblasts in a rabbit model. Ann Otol Rhinol Laryngol.

[94] Shin YS, Choi JW, Park J-K, Kim YS, Yang SS, Min B-H (2015). Tissue-engineered tracheal reconstruction using mesenchymal stem cells seeded on a porcine cartilage powder scaffold. Ann Biomed Eng.

[95] Walles T, Biancosino C, Zardo P, Macchiarini P, Gottlieb J, Mertsching H (2005). Tissue remodeling in a bioartifical fibromuscular patch following transplantation in a human. Transplantation.

[96] Hamilton N, Kanani M, Roebuck D, Hewitt R, Cetto R, Culme-Seymour E (2015). Tissue-engineered tracheal replacement in a child: a 4-year follow-up study. Am J Transplant.

[97] Wiszniewski L, Jornot L, Dudez T, Pagano A, Rochat T, Lacroix JS (2006). Long-term cultures of polarized airway epithelial cells from patients with cystic fibrosis. Am J Respir Cell Mol Biol.

[98] Skibinski G, Elborn JS, Ennis M (2007). Bronchial epithelial cell growth regulation in fibroblast cocultures: the role of hepatocyte growth factor. Am J Phys Lung Cell Mol Phys.

[99] Kobayashi K, Suzuki T, Nomoto Y, Tada Y, Miyake M, Hazama A (2010). A tissue-engineered trachea derived from a framed collagen scaffold, gingival fibroblasts and adipose-derived stem cells. Biomaterials.

[100] Mohd Heikal M, Aminuddin B, Jeevanan J, Chen H, Sharifah S, Ruszymah B (2010). Autologous implantation of bilayered tissue-engineered respiratory epithelium for tracheal mucosal regenesis in a sheep model. Cells Tissues Organs.

[101] Okano W, Nomoto Y, Wada I, Kobayashi K, Miyake M, Nakamura T (2009). Bioengineered trachea with fibroblasts in a rabbit model. Ann Otol Rhinol Laryngol.

[102] Delaere P, Vranckx J, Meulemans J, Vander Poorten V, Segers K, Van Raemdonck D (2012). Learning curve in tracheal allotransplantation. Am J Transplant.

[103] Delaere P, Vranckx J, Verleden G, De Leyn P, Van Raemdonck D (2010). Tracheal allotransplantation after withdrawal of immunosuppressive therapy. N Engl J Med.

[104] De Luca M, Albanese E, Cancedda R, Viacava A, Faggioni A, Zambruno G (1992). Treatment of leg ulcers with cryopreserved allogeneic cultured epithelium: a multicenter study. Arch Dermatol.

[105] Slonkova V, Navratilova Z, Semradova V, Adler J (2004). Successful treatment of chronic venous leg ulcers with lyophilized cultured epidermal allografts. Acta Dermatovenerol Alp, Pannonica, Adriat.

[106] Wu L, Leijten JC, Georgi N, Post JN, van Blitterswijk CA, Karperien M (2011). Trophic effects of mesenchymal stem cells increase chondrocyte proliferation and matrix formation. Tissue Eng A.

[107] Gray FL, Turner CG, Ahmed A, Calvert CE, Zurakowski D, Fauza DO (2012). Prenatal tracheal reconstruction with a hybrid amniotic mesenchymal stem cells–engineered construct derived from decellularized airway. J Pediatr Surg.

[108] Haykal S, Zhou Y, Marcus P, Salna M, Machuca T, Hofer SOP (2013). The effect of decellularization of tracheal allografts on leukocyte infiltration and of recellularization on regulatory T cell recruitment. Biomaterials.

[109] Haykal S, Salna M, Zhou Y, Marcus P, Fatehi M, Frost G (2014). Double-chamber rotating bioreactor for dynamic perfusion cell seeding of large-segment tracheal allografts: comparison to conventional static methods. Tissue Eng Part C: Methods.

[110] Butler CR, Hynds RE, Gowers KH, Lee DDH, Brown JM, Crowley C, et al. Rapid expansion of human epithelial stem cells suitable for airway tissue engineering. Am J Respir Crit Care Med. 2016;194(2):156–68. 10.1164/rccm.201507-1414OC PMC5003214 26840431

[111] Green H (2008). The birth of therapy with cultured cells. BioEssays.

[112] Lamichhane TN, Sokic S, Schardt JS, Raiker RS, Lin JW, Jay SM (2014). Emerging roles for extracellular vesicles in tissue engineering and regenerative medicine. Tissue Eng B Rev.

[113] Casey A, Dirks F, Liang OD, Harrach H, Schuette-Nuetgen K, Leeman K, et al. Bone marrow-derived multipotent stromal cells attenuate inflammation in obliterative airway disease in mouse tracheal allografts. Stem Cells Int. 2014;2014:11. 10.1155/2014/468927 PMC4177227 25295064

[114] Maughan EF, Butler CR, Crowley C, Teoh GZ, Den Hondt M, Hamilton N, et al. A comparison of tracheal scaffold strategies for pediatric transplantation in a rabbit model. Laryngoscope. 2017. 10.1002/lary.26611. 10.1002/lary.26611 28776693

[115] Bertolotti AM, Alvarez FA, Defranchi S, Alvarez M, Laguens RP, Favaloro RR (2012). Successful circumferential free tracheal transplantation in a large animal model. J Investig Surg.

[116] Seguin A, Baccari S, Holder-Espinasse M, Bruneval P, Carpentier A, Taylor DA (2013). Tracheal regeneration: evidence of bone marrow mesenchymal stem cell involvement. J Thorac Cardiovasc Surg.

[117] Close DM, Xu T, Sayler GS, Ripp S (2010). In vivo bioluminescent imaging (BLI): noninvasive visualization and interrogation of biological processes in living animals. Sensors.

[118] Pepper VK, Onwuka EA, Best CA, King N, Heuer E, Johnson J (2017). Endoscopic management of tissue-engineered tracheal graft stenosis in an ovine model. Laryngoscope.

[119] Guhad F (2005). Introduction to the 3Rs (refinement, reduction and replacement). J Am Assoc Lab Anim Sci.

[120] Hashemibeni B, Goharian V, Esfandiari E, Sadeghi F, Fasihi F, Alipur R (2012). An animal model study for repair of tracheal defects with autologous stem cells and differentiated chondrocytes from adipose-derived stem cells. J Pediatr Surg.

[121] Kilkenny C, Browne WJ, Cuthill IC, Emerson M, Altman DG (2010). Improving bioscience research reporting: the ARRIVE guidelines for reporting animal research. PLoS Biol.

[122] Weiss DJ, Elliott M, Jang Q, Poole B, Birchall M, Committee ISoCTPS (2014). Tracheal bioengineering: the next steps. Proceeds of an international society of cell therapy pulmonary cellular therapy signature series workshop, Paris, France, April 22, 2014. Cytotherapy.

[123] Komura M, Komura H, Kanamori Y, Tanaka Y, Suzuki K, Sugiyama M (2008). An animal model study for tissue-engineered trachea fabricated from a biodegradable scaffold using chondrocytes to augment repair of tracheal stenosis. J Pediatr Surg.

[124] Jungebluth P, Macchiarini P (2014). Airway transplantation. Thorac Surg Clin.

[125] Delaere P, Raemdonck DV (2016). Tracheal replacement. J Thorac Dis.

