# Supplementary material for: Autologous Cell Seeding in Tracheal Tissue Engineering
Source: Curr Stem Cell Rep. 2017 Oct 26;3(4):279–89. doi: 10.1007/s40778-017-0108-2 (PMC5683058; doi:10.1007/s40778-017-0108-2)
Supplement: Supplementary file 1 — (DOCX 35.7 kb). [file 40778_2017_108_MOESM1_ESM.docx]

Table 1: Reported *in vivo* orthotopic studies of cell seeding in tracheal TE

|  |  |  | **Cell seeding** | | | | | **Sample groups** | | **Implantation** | | **Results** | | | | |
| --- | --- | --- | --- | --- | --- | --- | --- | --- | --- | --- | --- | --- | --- | --- | --- | --- |
| **Author, Year** | **Species** | **Scaffold Strategy** | **External/ general cell type** | **Luminal cell type** | **Autolo-gous?** | **Seeding density** | **Seeding method** | **Experimental groups** | **Control group** | **Orthotopic/ heterotopic implantation** | **Timepoints (weeks)** | **Epithelia-lisation** | **Cartilage regene-ration** | **Vascular-isation** | **Mecha-nical failure** | **Sten-osis** |
| Co-seeding |  |  |  |  |  |  |  |  |  |  |  |  |  |  |  |  |
| Haykal *et al*, 2014 [109] | Pig | Decellularised - Matched pig trachea (DET - Triton & CHAPS) | MSC (bone marrow), labelled with CelltrackerGreen | Epithelial cells (trachea), labelled with CelltrackerOrange | y | 1,000,000/ml of each cell type | Static or dynamic seeding and culture in bioreactor for 72 hours | Scaffold with dynamic seeding (n=3) | Scaffold with static seeding (n=3) | Orthotopic | 12 |  |  |  | x |  |
| Tsao *et al*, 2014 [83] | Rabbit | Synthetic - PCL or PLGA porous casted rings, stented with piece of ET tube | Chondrocytes (auricular) | MSC (bone marrow) | n | total 2,000,000 cells/scaffold | co-seeded (Chondrocyte:BM-MSC 3:7) 1 week prior to implantation | 1. Orthotopic PCL scaffold + both cell types (2 weeks) (n=1); 2. Heterotopic PCL or PLGA scaffold + single cell type (4 weeks) (n=3); 3. Heterotopic PCL or PLGA + single cell type (2 weeks) (n=3) | 1. Heterotopic PCL or PLGA scaffold alone (4 weeks) (n=3); 3. Heterotopic PCL or PLGA alone (2 weeks) (n=3) | Heterotopic preimplantation in lateral thoaracic muscle flap, orthotopic transfer with intraluminal stent | 2,4 | x | x | x | x |  |
| Jungebluth *et al*, 2012 [84] | Pig | Decellularised - Matched pig trachea (DET - SDC & DNAse) | MSC (bone marrow) | Epithelial cells (trachea) | y | Per trachea = 32,000,000 MSCs; 1,390,000 ECs | Resuspended in autologous plasma prior to intraoperative seeding | Scaffold + cells (n=10) | None | Orthotopic | 8 | x | x |  |  |  |
| Go *et al*, 2010 [70] | Pig | Decellularised - Matched pig trachea (DET - SDC & RNAse) | Chondrocytes (bone marrow MSC-derived) | Epithelial cells (trachea) | y | ns | Dual-chamber continuously rotating bioreactor for 72 hours | 1. Scaffold + chondrocytes (n=5); 2. scaffold + epithelial cells (n=5); 3. scaffold with both cell types (n=5) | Scaffold alone (n=5) | Orthotopic | 8 | (x) |  |  | x | x |
| Kobayashi *et al*, 2010 [99] | Rat | Synthetic - Collagen sponge | MSC (adipose), labelled with mCherry | Fibroblasts (gingival), labelled with mYFP | y | 250,000/ml of each cell type | 3D culture in collagen gel for 3-4 weeks pre-implantation, on top of collagen sponge | 1. Scaffold with both cell types; 2. Scaffold + aMSCs; 3. scaffold + gingival fibroblasts. No mention of numbers | None | Orthotopic, anterior defect only | 1,2 | x |  |  |  |  |
| Mohd Heikal *et al*, 2010 [100] | Sheep | Synthetic - Autologous polymerised fibrin (bilayered) | Fibroblasts (nasal turbinates) | Epithelial cells (nasal turbinates) | y | 5,000,000 of each cell type/scaffold | Suspended in fibrin before polymerisation and immediate use | Scaffold + cells (n=6) | 1. Scaffold without cells (n=3); 2. Removal of mucosa only (n=3) | Orthotopic anterior mucosal graft only - titanium mesh temporary scaffold | 4 | x |  |  |  | x |
| Kojima *et al*, 2002 [76] | Sheep | Synthetic - PGA mesh wrapped in helix around silastic splint | Fibroblasts (nasal septum) | Chondrocytes (nasal septum) | y | 25,000,000 /ml fibroblasts; 50,000,000 /ml chondrocytes | Seeded into PGA mesh (internal chondrocyte helix, external fibroblast coating) before 1 week static culture | Scaffold + cells (n=6) | None | Heterotopic preimplantation under sternocleidomastoid muscle for 8 weeks before orthotopic free transfer | 1 |  | x |  | x | x |
| Luminal seeding only | |  |  |  |  |  |  |  |  |  |  |  |  |  |  |  |
| Wood *et al*, 2014 [87] | Dog | Decellularised - Size-matched canine trachea (freeze-thaw + detergent) | x | MSC (Adipose) | y | ns | Cells harvested, seeded in fibrin glue and implanted all under same anaesthetic | Scaffold + cells (n=4) | Scaffold alone (n=1) | Orthotopic | 12 |  |  |  | x |  |
| Okano *et al*, 2009 [93] | Rabbit | Synthetic - Polypropylene (mesh reinforced with spiral rings), coated with collagen sponge | x | Fibroblasts | n | 5000,000 /ml | Suspended in collagen and poured on sponge before gelation | Scaffold + cells (n=12) | Scaffold alone (n=12) | Orthotopic (anterior only) | 1, 2 | x |  |  |  |  |
| Kanzaki *et al*, 2006 [58] | Rabbit | Synthetic - Straight vascular prosthesis - knitted polyster, polypropylene spiral reinforcement | x | Epithelial cells (trachea) | ns | 200,000 cells/cm2 | Harvested as sheets from temperature-sensitive culture plates | Scaffold + cells (n=5) | Scaffold alone (n=5) | 4 weeks heterotopic graft placement (subcut). Cell sheets placed on luminal surface at time of transfer to orthotopic position. | 4 | x |  |  |  | x |
| General/external seeding only | | |  |  |  |  |  |  |  |  |  |  |  |  |  |  |
| Pepper *et al*, 2017 [118] | Sheep | Synthetic - Electrospun PET/PU with 3D polycarbonate rings | MSC (bone marrow) | x | y | ns | Vacuum seeded | Stent(s) when >50% luminal narrowing (n=6) | Dilation only (n=2) | Orthotopic | 3,6,12,16 |  |  |  |  | x |
| Clark *et al*, 2016 [57] | Sheep | Synthetic - Electrospun PET/PU with 3D polycarbonate rings | MSC (bone marrow) | x | y | 590,000,000 per graft - efficiency of 37-75% | Graft vacuum-seeded with bone marrow suspension immediately before surgery | Scaffold + cells (n=3) | Scaffold alone (n=2) | Orthotopic | 6 | (x) |  | x |  | x |
| Yan *et al*, 2016 [77] | Rabbit | Decellularized - rabbit trachea with or without synthetic - PLGA/PTMC | Chondrocytes (bone marrow MSC-derived) | x | y | 10,000,000/ml |  | Hybrid scaffold + cells (n=10); decellularised scaffold + cells (n=10) | none | Orthotopic with heterotopic subcut preimplantation with NG tube stent (2 weeks) | 4 |  | x |  |  |  |
| Batioglu-Karaaltin *et al*, 2015 [56] | Rabbit | Decellularised - Matched rabbit trachea (freeze-thaw then DET - SDC & RNAse) | MSC (Adipose) | x | y | Initially 8,000,000/ml, reseeded at 6,000,000/ml for last 24hrs | Static seeding for 24 hours then roller for 48 hours | Scaffold + cells (n=3) | Scaffold alone (n=3) | Orthotopic | 4, 8, 12 | x | x | x |  | x |
| Komura *et al*, 2015 [60] | Rabbit | Synthetic - 2-layer composites: gelatin sponge + β-tricalcium phosphate (TCP) + vicryl mesh, cross-linked with glutaraldehyde | Chondrocytes (auricular) | x | y | 10,000,000/ml | Extraluminal placement of chondrocytes in collagen sponge. | Scaffold + cells (n=5) | None | Heterotopic preimplantation in oesophagus. Orthotopic transfer to trachea at 4 weeks (posterior defect only) | 4 | x | x |  |  |  |
| Shin *et al*, 2015 [85] | Rabbit | Decellularised - Porcine articular cartilage (powder and reformed as gel) | MSC (bone marrow) | x | n | 150,000,000/ml | Implanted into gel for 7 weeks prior to implantation | Scaffold + cells (n=6) | None | Orthotopic (anterior only) | 6,10 | x | x |  |  |  |
| Chang *et al*, 2014 [61] | Rabbit | Synthetic - 3D-printed PCL | MSC (bone marrow) | x | n | 5,000,000/ml | *In vitro* coating of MSCs in fibrin | Scaffold + cells (n=4) | None | Orthotopic (anterior only) | 8 | x | x |  |  |  |
| Hong *et al*, 2014 [78] | Rabbit | Synthetic - Fibrin/Hyaluronan hydrogel / PGLA | Chondrocytes (auricular) | x | n | 1,000,000/ml | Cultured in Fibrin/Hyaluronan hydrogel, injected into PGLA scaffold for 4 weeks | Scaffold + cells (n=8) | None | Orthotopic (anterior only) | 6, 10 | x | x | x | x | x |
| Shin *et al*, 2014 [85] | Rabbit | Decellularised - Porcine articular cartilage (powder and reformed as gel) | Chondrocytes (articular) | x | y | 1,500,000/ml (total 600,000 cells/scaffold) | Implanted into gel for 7 weeks prior to implantation | Scaffold + cells (n=6) | None | Orthotopic (anterior only) | 2,4,8 | x | x |  | x |  |
| Kim *et al*, 2013 [79] | Rabbit | Decellularised - Matched rabbit trachea (freeze-thaw) | MSC (bone marrow) | x | n | 10,000,000 /kg body weight | Injected IV post-transplantation | 1. Scaffold + cells (n=8); 2. Scaffold + cells + ciclosporin (n=8) | 1. Scaffold + ciclosporin (n=8); 2; Scaffold alone (n=8) | Orthotopic with contemporaneous muscle wrap | 4 | x |  | x |  | x |
| Luo *et al*, 2013 [62] | Rabbit | Synthetic - Woven PGA wrapped around silicone stent | Chondrocytes (auricular) | x | y | 60,000,000/ml (1ml/scaffold) | Static seeding for 2 weeks | Scaffold + cells with intramusular (sternohyoid) pedicle (n=10) | Scaffold + cells with subcut implantation & subsequent free transfer (n=10) | Orthotopic with heterotopic preimplantation with silicone stent (4 weeks) |  | x | x | x | x |  |
| Nomoto *et al*, 2013 [80] | Rabbit | Synthetic - Collagen sponge with polypropylene frame | Chondrocytes (costal) | x | y | 2,000,000/ml | Suspended in collagen and poured on sponge before gelation | Scaffold + cells (n=10) | Scaffold alone (n not stated) | Orthotopic (anterior only) | 2,8,14 | (x) | x | x |  |  |
| Seguin *et al*, 2013 [116] | Rabbit | Transplant - Unmatched rabbit descending aorta (female to male) with silicone intraluminal stent | MSC (bone marrow), labelled with GFP | x | y | Not stated | Bone marrow transplantation of labeled MSCs 3 months prior to surgery | 1. Scaffold + bone marrow labeled MSC transplantation (n=12); 2. Scaffold + cell injections POD 0, 10 & 21 (n=12) | Scaffold alone (n=12) | Orthotopic | 4, 12, 24, 36, 52, 60, 72 | x | x |  | x |  |
| Gray *et al*, 2012 [107] | Sheep (fetal) | Decellularised - Rabbit trachea (DET - SDC & RNAse) | MSC (amniotic fluid), labelled with GFP | x | y | 1,000,000/ml (static), then 7,500,000/ml (dynamic) | Differentiated to chondrocytes, then seeding on scaffold in static, then dynamic, bioreactor for 96 hours | Scaffold + cells (n=7) | Scaffold alone (n=6) | Orthotopic (*in utero* at 115-122/145 gestation) | 1 week post-delivery (4-5 weeks total) | x |  |  |  | x |
| Hashemibeni *et al*, 2012 [120] | Dog | Synthetic - Alginate | MSC (Adipose), with or without differentiation to chondrocytes | x | y | 5,000,000/ml | Not stated | 1. Scaffold + undiff. MSCs (n=6); 2. scaffold + diff. chondrocytes (n=6) | Scaffold alone (n=6) | Orthotopic (anterior portions of cartilage rings only) - each animal had 3 ring defects with a different graft in each | 8 |  | x |  |  |  |
| Gilpin *et al*, 2010 [63] | Rabbit | Synthetic - Semipermeable polyester membrane precoated with fibronectin, several sheets stacked together | Chondrocytes (auricular) | x | y | 30,000,000/16cm2 bioreactor, reseeded with 12,000,000 at 7 days | Cultured for 7-8 weeks on sheets | Scaffold + cells (n=7) | None | LTR with anterior graft | 4, 8, 12 |  | (x) |  | x | x |
| Kim *et al*, 2010 [64] | Rabbit | Synthetic - Fibrin/Hyaluronan stabilised hydrogel | Chondrocytes (auricular) | x | y | 1,250,000 per scaffold | Statically incubated for up to 2 weeks | Scaffold + cells (n=6) | none | Orthotopic (anterior only) | 4, 8 |  |  |  |  |  |
| Lin *et al*, 2009 [86] | Rabbit | Synthetic - PCL with rings of collagen gel | Chondrocytes (articular) | x | n | 3,000,000 cells per collagen ring | Chondrocytes implanted in collagen prior to gelation - static culture for 7 days, then dynamic bioreactor for 4 or 8 weeks at different rotational speeds | Scaffold + cells (n=6) | None | Orthotopic | Until airway stenosis caused DIB | x | (x) | x |  | x |
| Weidenbecher *et al*, 2009 [65] | Rabbit | Scaffold-free' - 3 sheets of cells wrapped around silicone tube on table (muscle flap in between) | Chondrocytes (auricular) | x | y | Not stated | chondrocytes seeded as sheets in bioreactor for 4 weeks | 1. Scaffold with partial resection of intraluminal muscle flap portion (n=2); 2. Scaffold with partial resection of flap and delayed reconstruction (n=2); 3. Scaffold with complete resection of luminal flap (n=1) | Scaffold with intraluminal muscle flap intact (n=1) | Heterotopic implantation in sternohyoid muscle flap for 12-14 weeks before orthotopic transfer | until airway stenosis caused DIB |  | x | x |  | x |
| Komura *et al*, 2008 [123] | Rabbit | Synthetic - layered composites: gelatin/b-FGF microspheres + collagen sheet + PGA mesh + PLLA/PCL mesh | Chondrocytes (auricular) | x | y | Not stated | Static seeding onto scaffold 24hrs before implantation | Scaffold + cells + gelatin microspheres (n=10); Scaffold + cells (n=7) | none | Orthotopic (anterior only) | 4, 12 | x |  |  |  |  |
| Nomoto *et al*, 2008 [68] | Mouse | Synthetic - Collagen sponge | Fibroblasts (rat trachea), transfected with mYFP | x | n | 500,000/ml | Suspended in collagen and poured on sponge before gelation | Scaffold + cells (n=9) | 1. Acellular collagen + sponge (n=9); 2. Acellular sponge only (n=9) | Orthotopic (anterior only) | 3, 7, 14 days | x |  |  |  |  |
| Suzuki *et al*, 2008 [55] | Rat | Synthetic - Collagen sponge | MSC (Adipose), labelled with mYFP | x | y | 3,500,000/ml | 3D culture in collagen gel for 3-4 weeks pre-implantation, on top of collagen sponge | Scaffold + cells (n=20) | Scaffold alone (n=20). No mention of numbers | Orthotopic (anterior only) | 1,2 | x |  | x |  |  |
| Kunisaki *et al*, 2006 [66] | Sheep | Synthetic - PGA mesh rolled into cylinders, sprayed with PLA & collagen | MSC (amniotic fluid), labelled with GFP | x | n | 40-80,000,000/cm2 | Seeded on scaffold in static, then dynamic, bioreactor for 24-30 weeks for differentiation to chondrocytes | Scaffold + cells (n=7) | None | Orthotopic - anterior only (n=6), or full interposition graft (n=1) | 10 days | x | x |  |  | x |
| Grimmer *et al*, 2004 [81] | Rabbit | Synthetic - PGA constructs + RGD-modified alginate | Chondrocytes (auricular) | x | y | 1,000,000/ml | Cells harvested 24hours pre-op, seeded into liquid alginate, infused into PGA mesh | Complete scaffold + cells + PLLA intraluminal stent (n=5); Complete scaffold + cells (n=5) | Historical controls (fascia lata alone) (n=32) | Orthotopic (anterior only) with contemporaneous fascia lata wrap | 20 |  | x |  | x | x |
| Fuchs *et al*, 2003 [71] | Sheep (fetal) | Synthetic - PGA | Chondrocytes (Lamb BM-MSC derived OR fetal lamb elastic chondrocytes) | x | n | 60,000,000 /cm2 | Differentiated to chondrocytes pre-implantation on scaffold in dynamic bioreactor for 12 weeks | Scaffold + BM-MSC (n=4) | Scaffold + chondrocytes (n=4) | Orthotopic (anterior only, *in utero* at 110-120/145 gestation) | At delivery (4 weeks total) | x | x |  |  | x |
| Lee *et al*, 2002 [67] | Rabbit | Synthetic - PLGA, 80% porosity | Chondrocytes (costal) | x | n | Not stated | 24 hr dynamic seeding, then 48 hr static incubation | Scaffold + cells (n not stated) | None | Orthotopic (anterior only) | 6 | x | x |  |  |  |
| Fuchs *et al*, 2002 [69] | Sheep (fetal) | Synthetic - PLLA over PGA mesh with collagen coating | Chondrocytes (elastic auricular or hyaline tracheal) | x | y | 40-80,000,000/cm2 | 24hrs static seeding then 6-8 weeks rotating culture | Scaffold + cells (n=15) | Elastic cartilage from autologous ear (n=5) | Orthotopic (anterior only) | 1, 2, 2.5, 3 and at birh | x | x | x | (x) |  |
