# Supplementary material for: Autologous Cell Seeding in Tracheal Tissue Engineering
Source: Curr Stem Cell Rep. 2017 Oct 26;3(4):279–89. doi: 10.1007/s40778-017-0108-2 (PMC5683058; doi:10.1007/s40778-017-0108-2)
Supplement: Supplementary file 2 — (DOCX 86.9 kb). [file 40778_2017_108_MOESM2_ESM.docx]

Table 2: Reported clinical cases of tracheal TE involving autologous cell seeding

| **General** | |  | **Cell seeding** | | | **Implantation** | | | | **Results** | | | | |
| --- | --- | --- | --- | --- | --- | --- | --- | --- | --- | --- | --- | --- | --- | --- |
| **Age/Sex** | **Clinical background** | **Scaffold Strategy** | **General/ external surface** | **Luminal surface** | **Seeding method** | **Surgical technique** | **Follow-up** | **Post-op interventions** | **Clinical outcome** | **Epithelia-lisation** | **Cartilage regenera-tion** | **Vascula-risation** | **Malacia/stent required** | **Steno-sis** |
| **Omori Group (Fukushima) [88-90]** | | | | | | | | | | | | | | |
| 78F | Thyroid cancer invading trachea | Synthetic | Venous blood | x | Blood injected into collagen at time of operation | Single-stage partial circumference tracheal resection and graft placement | 34m | Bronchoscopy (2w, 2m, 7m, 20m) | Patent unassisted airway | y |  |  | n | n |
| 59F | Thyroid cancer invading trachea | Synthetic | Venous blood | x | Blood injected into collagen at time of operation | Single-stage partial circumference tracheal resection and graft placement | 12m | Bronchoscopy (1w, 2m, 12m) | At 2 months, more than half the graft covered by epithelium. Patent unassisted airway | y |  |  |  |  |
| 71M | Thyroid cancer invading trachea | Synthetic | Venous blood | x | Blood injected into collagen at time of operation | Single-stage partial circumference tracheal resection and graft placement | 2m | Bronchoscopy (2w, 2m, 22m) | Patent unassisted airway | y |  |  | n | n |
| 77M | Subglottic stenosis secondary to tracheostomy closure | Synthetic | Venous blood | x | Blood injected into collagen at time of operation | Two-stage graft placement at 4m in defect created by previous resection of stenotic segment | 8m | Bronchoscopy (2w, 2m, 8m) | Some granulation tissue at 2months. Patent unassisted airway | y |  |  | n | y (mild) |
| 39F | Subglottic/tracheal stenosis (Intubation for status asthmaticus) | Synthetic | Venous blood | x | Blood injected into collagen at time of operation | Two-stage graft placement at 4m in defect created by previous resection of stenotic segment | 6m | Bronchoscopy, CT | Necrosis of long segment of trachea. Patent unassisted airway | y |  |  | n | y (mild) |
| 45M | Subglottic & laryngeal stenosis (Intubation for inhalation burn) | Synthetic | Venous blood | x | Blood injected into collagen at time of operation | Two-stage graft placement at 4m in defect created by previous resection of stenotic segment | 6m | Bronchoscopy, CT | Patent unassisted airway | y |  |  | n | n |
| 71F | Subglottic/tracheal stenosis (Intubation for road traffic accident) | Synthetic | Venous blood | x | Blood injected into collagen at time of operation | Two-stage graft placement at 4m in defect created by previous resection of stenotic segment | 6m | Bronchoscopy, CT | Patent unassisted airway | y |  |  | n | n |
| **Macchiarini Group (Karolinska/UCL) [14,35,82,124]** | | | | | | | | | | | | | | |
| 30F | End-stage malacia of left main bronchus 2o to TB | Decellularised | Chondrocytes (bone marrow MSC-derived) | Epithelial cells (right main bronchus) | Dual-chamber bioreactor (static seeding of each quadrant of lumen for 30mins), continuously rotating for 96 hours | Full circumference replacement, no vascularisation flap | 5yr | Bronchoscopy (4d, 2w, 1m, 2m, 3m, then every 3m); Laser-doppler; anti-HLA serology. Lung function tests; Quality of life scores; CT | Patent airway at 4 months. Stenosis of proximal anastomosis - critical stenosis (12m), graft stenting (14m). Graft & lung removed 2016 (7yr). | y |  | y | n |  |
| 36M | Recurrent tracheobronchial mucoepidermoid carcinoma, post-radiotherapy & debulking | Synthetic | MSC (Bone marrow) | x | bioreactor, continuously rotating for 36 hours, reseeded immediately prior to implantation. | Resection of tumour and involved structures, full circumferential replacement of trachea & bronchi with omental wrap, temporary tracheostomy for graft cleaning. | 5m | Bronchoscopy (daily for first week, weekly for rest of admission, then monthly) | Patent unassisted airway at 5months, report alive with patent airway at 24m. Media reported deceased at 31m post-implantation |  |  | y |  | y (mild) |
| **Walles group (Würzburg) [91,92,95]** | | | | | | | | | | | | | | |
| 58M | Anastomotic defect at tracheobronchial anastomosis (carinal pneumonectomy for NSCLC following post-radiotherapy relapse) | Decellularised | x | Skeletal muscle cells (SMC) + Fibroblasts (Fb) 5:95 | Luminal surface seeded statically for 3w | Single-stage membranous tracheal reconstruction following closure of oesophageal fistula, omental transposition flap | 12w | Bronchoscopy (1w, 3w, 6w, 12w) | |  |  |  |  |  |
| 26M | Extensive distal tracheoesophageal fistula following caustic ingestion | Decellularised | Microvascular endothelial cells (mvEC) | Skeletal muscle cells (SMC) | mvEC perfused via vasulature for 5 days, then SMC applied to lumen for 5 days | Single-stage membranous tracheal reconstruction following closure of oesophageal fistula, no vascularisation/interposition flap | 2.5yr | Bronchoscopy (8d, 2w, 2.5yr) | Eating & drinking normally, able to play soccer | y | y |  | n | n |
| **Sumitran-Holgersson group (Gothenburg) (Berg et al, 2013 - RETRACTED)** | | | | | | | | | | | | | | |
| 76M | Long tracheal stenosis & malacia (Intubation for trauma) | Decellularised | Chondrocytes (bone marrow MSC-derived) | Epithelial cells (nasal mucosa) | Dual-chamber bioreactor (static seeding of each quadrant of lumen for 60mins), continuously rotating for 2w. | Full circumference replacement, no vascularisation flap | 23d | Flexible bronchscopy (3d, 8d, 20d); anti-HLA/endothelial ell serology. | Patient died of cardiac arrest POD23 - patent airway at PM | n | n |  | n | n |
| **Delaere group (Leuven) [102,103,125]** | | | | | | | | | | | | | | |
| 55F | Tracheal stenosis (Intubation for road traffic accident) | Transplant | N/A | Buccal mucosa | Immunosuppression withdrawn once viable recipient cells detected within grafts (7.5m post-implant) | Circumferential 4.5cm donor tracheal segment transferred as radial forearm composite free flap, 9m post-implant | 1yr | CT | Patent unassisted airway. Necrosis of membranous trachea over first 4 weeks of forearm implantation (removed at time of buccal mucosa graft). | y | y | y | n | n |
| 26M | Tracheal stenosis (Intubation for inhalation burn) | Transplant | N/A | Buccal mucosa | Immunosuppression withdrawn after 4m | - | N/A | CT | Rejection of graft 2w after cessation of immunosuppression, not transplanted to airway | n | n | n |  |  |
| 45F | Tracheal stenosis (Traumatic intubation) | Transplant | N/A | Buccal mucosa | Immunosuppression withdrawn 1m after orthotopic tissue transfer | Circumferential 6cm donor tracheal segment transferred as radial forearm composite free flap, 2m post-implant | 5m | CT | Donor mucosal necrosis following immunosuppression cessation, followed by graft stenosis | y | y | y |  | y (mild) |
| 17M | Tracheal stenosis (traumatic intubation) | Transplant | N/A | Buccal mucosa | Immunosuppression withdrawn 1m after orthotopic tissue transfer | Circumferential 6cm donor tracheal segment transferred as radial forearm composite free flap, 2m post-implant | 5m | CT | Donor mucosal necrosis following immunosuppression cessation, followed by graft stenosis. Required tracheostomy. | y | y | y |  | y |
| 64M | Chronic tracheal chondrosarcoma | Transplant | N/A | Buccal mucosa | Graft denuded of donor mucosa prior to buccal grafts, 2m post-implant | Circumferential 6cm donor tracheal segment transferred as radial forearm composite free flap, 5m post-implant | 6m | CT | Patent unassisted airway without tumour recurrence (immunosuppression phased out between 15-18m post-implant) | y | y | y |  | n |
| **Elliott group (Great Ormond Street) [15,16,59]** | | | | | | | | | | | | | | |
| 12M | Congenital long-segment tracheal stenosis with erosion of metal airway stent into aorta | Decellularised | MSC (Bone marrow) | Epithelial cells (tracheal mucosa) | Graft saturated with cell suspension and growth factors intra-op, mucosal stamps applied as free luminal grafts intra-op | Full circumferential replacement on CP bypass, wih PDS stent | 5y | Bronchoscopy (frequent for first 6 months, then spaced out). 1 further balloon dilatation required to distal trachea & untransplanted left main bronchus in 3rd post-operative year. | Patent airway, back at school, no medical interventions required for 6 months. 3 further admissions to ICU in 2nd year, but back in full-time education. Free of medical intervention for long periods. | y | y | y | y | y (mild) |
| 15F | Congenital long-segment tracheal stenosis, single lung, failed previous reconstructions & stenting | Decellularised | MSC (Bone marrow) | Epithelial cells (nasal mucosa) | Seeding in bioreactor | Full circumferential replacement on CP bypass | 15d | Bronchoscopy (1d, 7d) | Able to speak in full sentences and eat & drink normally within 24hrs. Death on POD15 under anaesthesia (prolonged respiratory arrest), PM declined by family. |  |  |  |  |  |
